# Simulating a base population in honey bee for molecular genetic studies

**DOI:** 10.1186/1297-9686-44-14

**Published:** 2012-06-27

**Authors:** Pooja Gupta, Tim Conrad, Andreas Spötter, Norbert Reinsch, Kaspar Bienefeld

**Affiliations:** 1 Institute for Bee Research Hohen Neuendorf, 16540, Hohen Neuendorf, Germany; 2 Institute of Mathematics, Freie Universitaet Berlin, Berlin, Germany; 3 Leibniz Institute for Farm Animal Biology, 18196, Dummerstorf, Germany

## Abstract

**Background:**

Over the past years, reports have indicated that honey bee populations are declining and that infestation by an ecto-parasitic mite (*Varroa destructor*) is one of the main causes. Selective breeding of resistant bees can help to prevent losses due to the parasite, but it requires that a robust breeding program and genetic evaluation are implemented. Genomic selection has emerged as an important tool in animal breeding programs and simulation studies have shown that it yields more accurate breeding value estimates, higher genetic gain and low rates of inbreeding. Since genomic selection relies on marker data, simulations conducted on a genomic dataset are a pre-requisite before selection can be implemented. Although genomic datasets have been simulated in other species undergoing genetic evaluation, simulation of a genomic dataset specific to the honey bee is required since this species has a distinct genetic and reproductive biology. Our software program was aimed at constructing a base population by simulating a random mating honey bee population. A forward-time population simulation approach was applied since it allows modeling of genetic characteristics and reproductive behavior specific to the honey bee.

**Results:**

Our software program yielded a genomic dataset for a base population in linkage disequilibrium. In addition, information was obtained on (1) the position of markers on each chromosome, (2) allele frequency, (3) χ^2^ statistics for Hardy-Weinberg equilibrium, (4) a sorted list of markers with a minor allele frequency less than or equal to the input value, (5) average r^2^ values of linkage disequilibrium between all simulated marker loci pair for all generations and (6) average r^2^ value of linkage disequilibrium in the last generation for selected markers with the highest minor allele frequency.

**Conclusion:**

We developed a software program that takes into account the genetic and reproductive biology specific to the honey bee and that can be used to constitute a genomic dataset compatible with the simulation studies necessary to optimize breeding programs. The source code together with an instruction file is freely accessible at http://msproteomics.org/Research/Misc/honeybeepopulationsimulator.html

## Background

The honey bee is an economically important species that serves as a major pollinator of wild plants and agricultural crops. Over the past years, a decline of honey bee populations has been reported [1-3] mainly caused by infestation with an ecto-parasitic mite (*Varroa destructor*). Selective breeding of resistant bees can help prevent losses due to the parasite, but it requires that a robust breeding program and genetic evaluation are implemented. Until now, the approach used in honey bee is a traditional breeding program based on the best linear unbiased prediction (BLUP) relying on pedigree data [4]. Recently, genomic selection strategies based on molecular marker data have emerged as a promising approach [5]. Marker-based selection has been widely tested in several species either with simulated datasets e.g. [6,7] or with real datasets e.g. [8-11] but, to date, not in honey bee. Since simulation studies require molecular genetic and pedigree datasets to ascertain selection methods, our primary aim was to develop a software program capable of producing a dataset for a base population in the honey bee and which could be used to implement marker-based selection procedures. A base population is used as a starting point for simulation studies. It is composed of individuals in a pedigree for which no ancestral information is available, and is assumed to be in linkage disequilibrium (LD). The second goal of our software program was to provide the user with the possibility to investigate the effect of parameters like mutation rate, density of markers and number of individuals on the extent of LD in a population.

## Implementation

Honey bee has specific genetic and reproductive characteristics, such as a high recombination rate, haplo-diploid sex determination, arrhenotoky and polyandry, which require appropriate software for modeling. The features of our software program and the modeling of population structure, genome and evolutionary processes are described below. In order to simulate a dataset according to the requirements specific to honey bee populations, it is necessary to design a software program that will allow to input: (1) number of generations, (2) number of sire queens, (3) number of dam queens, (4) number of marker loci, (5) forward and backward mutation rates, (6) minor allele frequencies and (7) number of marker loci to be selected as SNP (single nucleotide polymorphism) on the basis of minor allele frequency. The MATLAB source code and an instruction file on how to use the software program can be found on its website [12].

## Setting up of the population structure

A population can be structured according to the provided input. The input data include number of sire queens and dam queens (with a ratio of 10:1), number of generations and total number of marker loci to be simulated (assumed to be bi-allelic). In order to model the haploid drones, a sire queen is defined as representing a drone-producing colony. Two matrices with a size equal to number of individuals by number of marker loci represent the genome of diploid sire queens and dam queens, respectively. The population size is kept constant in every generation according to the Fisher-Wright population model. Furthermore, all simulated generations are non-overlapping.

## Genome simulation

A diploid genome, consisting of 16 linkage groups, is simulated for sire and dam queens. The length of each chromosome is simulated according to the actual length of all honey bee chromosomes. The number of marker loci (*N*) to be simulated along the genome can be provided as input. In the software program, the number of marker loci to be distributed per chromosome, *N*_*i*_ (*i* = 1, 2, .....16), is based on the actual proportion of SNP loci present on each honey bee chromosome and is computed using the following formula:

(1) $$\text{Number of marker locion the }i^{\mathrm{th}}\text{ chromosome},N_{i}=NR_{i}$$

where *R*_*i*_ is equal to the actual ratio between number of SNP loci on the *i*^*th*^ chromosome and total number of SNP loci in the honey bee genome. Positions of all the loci on all chromosomes are sampled from a uniform distribution. The number of SNP loci per chromosome and the length of all 16 chromosomes were obtained from the honey bee genome database [13,14] as shown in Table 1.

**Table 1:** Summary of the simulated chromosome length, number of SNP and R_i_

**Chromosome**	**Length (in base-pairs)**	**Number of SNP**	**R**_**i**_
1	29893408	140148	0.1414
2	15549267	62801	0.0633
3	13234341	70577	0.0712
4	12718334	55407	0.0559
5	14363272	62750	0.0633
6	18472937	78086	0.0788
7	13219345	59210	0.0597
8	13546544	61811	0.0623
9	11120453	55302	0.0558
10	12965953	50243	0.0507
11	14726556	68972	0.0696
12	11902654	57616	0.0581
13	10288499	50380	0.0508
14	10253655	48322	0.0487
15	10167229	38452	0.0388
16	7207165	31295	0.0316
Total	219629612	991372	

Chromosome length and SNP data were obtained from the honey bee genome database; R_i_ is the actual ratio between the number of SNP on the i^th^ chromosome and the total number of SNP in the honey bee genome; this information is used to simulate the genome and distribute markers across the chromosomes

## Evolution simulation

To simulate an evolutionary process, recombination and mutation are implemented during the process of gamete formation in every generation. Multiple mating is modeled in the parental generations. The processes are briefly described below.

## *Recombination*

Recombination is the exchange of chromosomal segments between paternal and maternal chromosomes and is implemented as follows. The recombination probability (*θ*) between two adjacent loci on a chromosome is calculated from the Haldane mapping function [15], which is the most widely used mapping function. It is based on the assumption that crossovers in any given chromosomal segment follow a Poisson distribution, with no interference between crossovers. In the software program, the recombination probability is calculated using the following expression:

(2) $$\theta =\frac{1}{2}\left[1-\exp\left(-2|x|\right)\right]$$

where exp denotes the exponential function and |*x*| stands for the absolute value of the map distance between adjacent loci. The Haldane mapping function requires that distances are expressed in Morgan units, therefore, distances between adjacent loci are converted from base-pairs to Morgan using the reported recombination rate of 19 cM/Mb [16,17].

## *Mutation*

Mutation is implemented in our software program to create polymorphisms. All loci in all individuals belonging to generation zero have a single allele coded as 1. We model both forward and backward mutations, allowing each locus to mutate from allele 1 to allele 2 and from allele 2 to allele 1. The required rates of forward and backward mutations can be specified in the input as mutation rates per locus per gamete per generation. The advantage of modeling a bi-directional mutation is that different values of forward and backward mutation rates can be chosen. Setting the backward mutation rate to zero will result in an infinite site model of mutation [18] where each locus can only mutate once in the entire generation and mutation will result in the formation of allele 2. The infinite site model of mutation can be useful when simulating an extremely high initial marker density with a low mutation rate and larger number of generations as shown in studies by Sorrenson and Meuwissen [6] and Calus et al. [19], where 1 million and 300 000 marker loci were simulated for a genome of size 10 M and 3 M, respectively.

## *Multiple mating*

**Figure 1:**
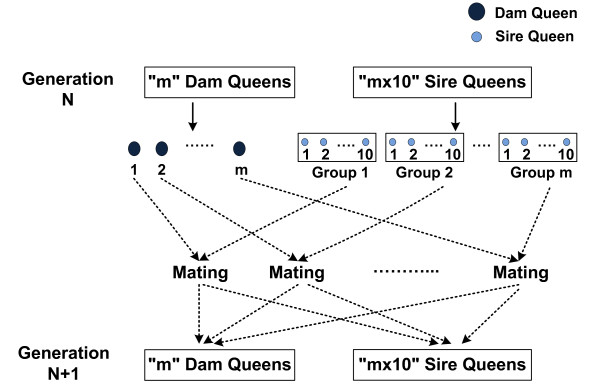
**General mating scheme.** m = total number of dam queens; since there is a 1:10 ratio between number of dams and sires, the number of sire queens is “mx10”; in every generation, all sire queens are randomly permuted and grouped; each group consists of 10 sire queens; a dam queen and a group of sire queens are the mating partners; all generations are non-overlapping and the population size is kept constant across generations.

Figure 1 describes the general mating scheme followed during the simulation. Polyandry, commonly referred to as multiple mating, is a phenomenon observed in honey bee whereby a queen mates with multiple drones (average of 10 to 20 drones). To model this situation in our software program, a dam queen and a group of 10 sire queens (a sire queen represents a drone-producing colony) are assumed as mating partners. To form groups, all sire queens are randomly permuted and thereafter divided into groups consisting of 10 sire queens.

**Figure 2:**
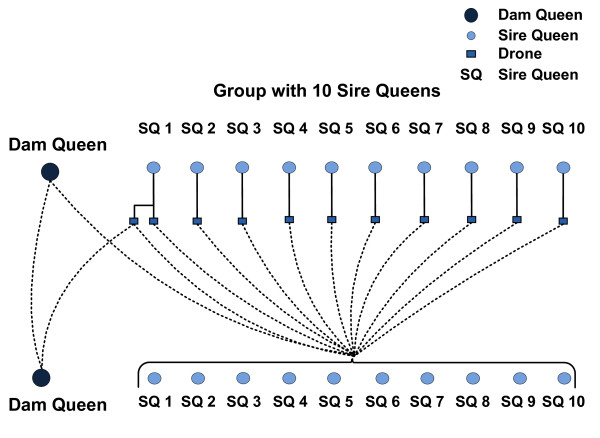
**Multiple mating between a dam queen and 11 drones from a group.** The resulting offspring consist of one dam queen and 10 sire queens; all drones are sampled with replacement which models the phenomenon of producing multiple copies of identical gametes by a drone.

A detailed mating scheme, showing how gametes from a dam queen are combined with the drones from a group of sire queens is illustrated in Figure 2. A dam queen generates a total of 11 gametes, of which 10 give rise to sire queens and one a dam queen in the next generation. Since a gamete produced by a sire queen is regarded as a drone, it is assumed to occur in multiple copies. One of the 10 sire queens of a group contributes a drone, which combines with a gamete from the dam queen to produce a new dam queen for the next generation. In addition to the drone generated for the formation of a dam queen, each of the 10 sire queens of a group produces one drone, thus a group contributes a total of 11 drones. During the formation of a sire queen, all 11 drones of a group have an equal probability to be drawn as a gamete. Since drones in a set are sampled with replacement, the resulting progenies are related as super-sibs (coefficient of relatedness = 0.75), full-sibs (coefficient of relatedness = 0.5) or half-sibs (coefficient of relatedness = 0.25).

## Statistics

The software program provides statistics for allele frequency, Hardy-Weinberg equilibrium, minor allele frequency and LD, which allow inferences to be made about an evolving population. Allele frequency data is a requisite for any population; hence the software program creates an output with allele frequencies for all loci in the base population along with the output for Hardy-Weinberg equilibrium and minor allele frequency, which are the usual criteria to evaluate marker loci informativeness. Most studies based on genome-wide marker data rely on the assumption that a marker and the locus affecting the trait are in LD, therefore, in the last generation, the average LD value is calculated for selected SNP with the highest minor allele frequency. In addition, generation-wise LD values for all simulated marker loci pairs are calculated and plotted on a graph. As a measure of LD, we used *r*^2^[20,21] which was calculated as follows:

(3) $$r^{2}=\frac{D^{2}}{f(A_{1})f(A_{2})f(B_{1})f(B_{2})}$$

where *D* = *f *(*A*_1_*B*_1_)*f *(*A*_2_*B*_2_) − *f *(*A*_1_*B*_2_)*f *(*A*_2_*B*_1_) and *f *(*A*_1_*B*_1_), *f *(*A*_2_*B*_2_), *f *(*A*_1_*B*_2_), *f *(*A*_2_*B*_1_), *f *(*A*_1_), *f *(*A*_2_), *f *(*B*_1_), *f *(*B*_2_) are observed frequencies of haplotypes A_1_B_1_, A_2_B_2_, A_1_B_2_, A_2_B_1_ and of alleles A_1_, A_2_, B_1_, B_2_ respectively in the population.

## Results and discussion

The software program developed in this study produces the following output: (1) the position of marker loci in base-pairs on each chromosome, (2) genotypes of all simulated individuals in the last generation, which can be used as a base population in further studies, (3) average r^2^ values of LD for all simulated marker loci pair for every simulated generation as a plot as well as a text file, (4) average r^2^ value of LD for the selected SNP with the highest minor allele frequency in the last generation, (5) allele frequencies for all loci in the base generation, (6) χ^2^ statistics for Hardy-Weinberg equilibrium for all loci in the base generation and (7) a list of marker loci with a minor allele frequency smaller than or equal to the input value.

## Example

To provide an illustration, we performed a run as example with the following input parameters: 

Number of generations: 2000

Number of sire and dam queens: two population sizes were simulated; the first one consisted of 500 sire and 50 dam queens and the second one of 200 sire and 20 dam queens.

Total number of marker loci: 100 000

Number of marker loci to be selected as SNP: 44 000

Forward and backward mutation rates: 0.0025

Minor allele frequency: 0.05

The values of mutation rate, recombination rate, marker density and effective population size determine both the level of LD in a population and the number of generations required to reach a certain value of LD. For the dataset example, the chosen mutation rate was set at 0.0025, a value similar to that used by Meuwissen et al. [5], allowing a high probability of polymorphic marker loci. Information on the level of recombination and on the effective population size (*N*_*e*_) in honey bee was obtained from Beye et al. [17] and Estoup et al. [22], respectively.

**Figure 3:**
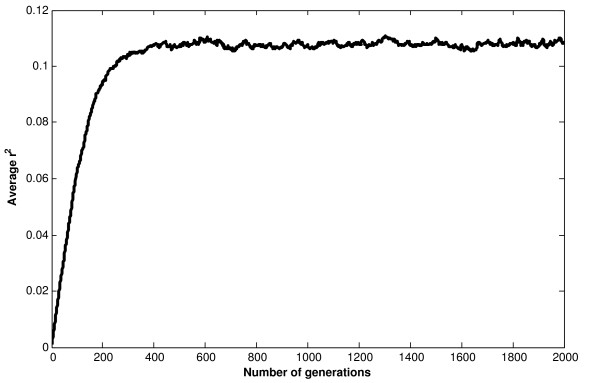
**The average value of r**^**2**^**plotted against number of generations for the input parameter values.** Simulation was performed for 2000 generations with a forward and backward mutation rate of 0.0025 for 100 000 marker loci and 220 colonies (20 dam queens and 200 sire queens); with the parameter values chosen here, a stable LD is reached after random mating.

All data and result files for this example can be found at the project home page [12]. In this paper, we only present the output graph (Figure 3) showing the establishment of LD for the simulated dataset with 220 queens. The graph shows the average LD reached in each generation and was obtained by calculating the average of LD value between all simulated pair of marker loci across the genome, not preselected on the basis of minor allele frequency.

In addition, we compared the expected average LD to the achieved average LD for 44 000 loci with the highest minor allele frequencies, which were chosen as SNP. The expected average LD in a population was calculated as follows [21]:

(4) $$r^{2}=\frac{5+2N_{e}c}{11+26N_{e}c+8N_{e}^{2}c^{2}}$$

where c is the recombination fraction between adjacent loci and *N*_*e*_ is the effective population size. Since the total size of the simulated genome was 219 629 612 base-pairs (Table 1) and the approximate recombination rate was taken as 19 cM/Mb [16,17], the size of the simulated genome was 41.73 M. Thus, for a genome of 41.73 M, c was approximately 0.001 for 44 000 SNP. The honey bee population has a wide range of effective population sizes [22]; therefore, we simulated two scenarios, one with 220 queens and other with 550 queens, respectively. The effective population size in the honey bee was calculated using the following expression for a haplo-diploid population [23,24]:

(5) $$N_{e}=\frac{9N_{f}N_{m}}{2\left(2N_{m}+N_{f}\right)}$$

where *N*_*f*_ is the number of queens (which is equal to the number of colonies since each colony is headed by a single queen) and *N*_*m*_ is the number of males. In the simulation, we assume that each queen is inseminated by 11 drones, therefore *N*_*m*_ = 11*N*_*f*_. Thus, with 220 and 550 colonies, *N*_*e*_ was approximately 473 and 1184, respectively. With *N*_*e*_ = 473, the expected LD was 0.24 and the achieved LD was 0.23. Similarly, with *N*_*e*_ = 1184, the expected and achieved LD were equal to 0.14 and 0.11, respectively. These values show that our software program is able to model the honey bee population with good accuracy.

Creating a dataset for a base population is a prerequisite for any simulation study, but it can be time consuming and it requires testing of optimum parameters. In most of the available software used to simulate populations [25], base population simulation is the preliminary stage, and is done by allowing the population to evolve through a burn-in period till the population reaches equilibrium from a random or uniform initial state. In our software program, all individuals in the starting generation are assumed to be unrelated. To establish LD, random mating is performed for the required number of generations and the final generation, which is in mutation-drift equilibrium, is taken as the base population. In genomic selection studies, a base population is the common starting point from which a population evolves further according to specific study requirements. To the best of our knowledge, this is the first software program that deals with evolutionary aspects in honey bee. It aims at providing an impetus to simulation studies in honey bee. It is an important initiative and we are developing strategies to simulate other honey bee datasets that could be used to implement genomic selection and furthermore to extend the study with a real genotyping dataset obtained from the SNP assay developed for honey bee [26]. The code is written in MATLAB but can be easily adapted to the open source version Octave.

## Conclusions

Our software program can construct a base population in LD by simulating a random mating honey bee population given some input population parameters. The statistics relevant to a population such as allele frequency, r^2^ value for LD and data for marker sorting according to minor allele frequency and Hardy-Weinberg equilibrium are provided in output files. The software program is relevant for research requiring a simulated molecular genetic honey bee dataset such as studies aiming at optimizing honey bee breeding programs.

## Availability and requirements

Project name: Honey Bee Population Simulator

Project home page: http://msproteomics.org/Research/Misc/honeybeepopulationsimulator.html

Operating system(s): Platform independent

Programming language: MATLAB

Other requirements: Tested for MATLAB version 7.9.0.529 (R2009b) and higher

License: The source code is available free of charge

Any restrictions to use by non-academics: none

## Abbreviations

BLUP = Best Linear Unbiased Prediction; LD = Linkage Disequilibrium; SNP = Single Nucleotide Polymorphism.

## Competing interests

The authors declare that they have no competing interests.

## Authors' contributions

PG wrote the manuscript and developed the software program. AS and TC participated in discussions and helped to draft the manuscript. NR and KB conceived the study, participated in discussions and helped to draft the manuscript. All authors read and approved the final manuscript.
